# Performance Assessment of Graphene Oxide as a Protective Coating for Historical Stone

**DOI:** 10.3390/ma18061243

**Published:** 2025-03-11

**Authors:** Codrut Costinas, Liviu Cosmin Cotet, Lucian Baia, Naida El Habra, Luca Nodari, Patrizia Tomasin

**Affiliations:** 1 Faculty of Physics, Babeș-Bolyai University, M. Kogălniceanu 1, RO-400084 Cluj-Napoca, Romania; codrut.costinas@ubbcluj.ro (C.C.); lucian.baia@ubbcluj.ro (L.B.); 2 Laboratory for Advanced Materials and Applied Technologies, Institute for Research, Development and Innovation in Applied Natural Sciences, Babeș-Bolyai University, Fântânele 30, RO-400294 Cluj-Napoca, Romania; 3 Faculty of Chemistry and Chemical Engineering, Babeș-Bolyai University, Arany Janos 11, RO-400028 Cluj-Napoca, Romania; 4 CNR-ICMATE (Istituto di Chimica della Materia Condensata e di Tecnologie per l’Energia), Corso Stati Uniti 4, 35127 Padova, Italy; naida.elhabra@cnr.it (N.E.H.); luca.nodari@cnr.it (L.N.)

**Keywords:** graphene oxide, stone cultural heritage, protective coatings, weathering, water capillary absorption, water vapor permeability

## Abstract

Stone cultural heritage faces significant deterioration from environmental factors, necessitating protective treatments that preserve both functionality and appearance. In this study, graphene oxide (GO) was evaluated as a protective coating for both natural and artificially aged Euganean trachyte and Vicenza stone samples. GO was applied as a low-concentration aqueous dispersion (0.5 mg/mL) by brush, and samples were subsequently exposed to UV light for 7 h to simulate weathering. Performance was assessed in accordance with European standards through measurements of water capillary absorption, water vapor permeability, contact angle, and color variation; further characterization was conducted using FT–IR, Raman spectroscopy, SEM, and XRD. Results indicate that GO coatings reduce the water capillary absorption coefficient by up to 49% for Euganean trachyte and 22% for Vicenza stone, while maintaining vapor permeability close to that of untreated samples. Although UV exposure permanently darkens the coating, it slightly enhances hydrophobicity, likely due to differential photoreduction of thin surface layers versus thicker pore-associated GO domains. These findings suggest that, while GO, particularly after UV weathering, shows promise for stone protection, further research is crucial to optimize coating uniformity and assess long-term durability under realistic environmental conditions.

## 1. Introduction

Stone, the building block of a large number of historical buildings, is subject to natural or anthropic deterioration processes, which could threaten the integrity and sometimes the stability of constructions. Stone deterioration is the result of the simultaneous action of physical, mechanical, chemical or biological agents [1], and its rate has increased from the twentieth century onwards due to the increasing concentration of atmospheric pollutants, such as carbon derivatives, sulfur dioxide, or nitrogen oxide and particulate matter, especially in the urban environments [2,3]. Water plays a crucial role in many decay mechanisms, often considered the most significant deteriorating agent. It drives numerous degradation processes, including freeze–thaw cycles, salt crystallization, and the diffusion of contaminants within the stone’s microstructure [1]. Furthermore, water and humidity promote the growth of microorganisms, and the resulting biofilms can exacerbate deterioration [1]. Climate change further threatens our built cultural heritage. Altered rainfall patterns, increased flood risk, and other extreme weather events present additional challenges to the preservation of stone monuments [4,5].

Protective coatings are commonly used with the goal of preventing degradation processes that occur mainly at the interface with the environment, by reducing the permeation of water, pollutants or salts inside the stone or minimizing biodegradation through their antimicrobial properties [6,7]. In many cases, the treatment is chosen for a specific purpose dictated by the largest risks of damaging the stone substrate, and can be water repellent, anti-graffiti, salt inhibitors, anti-fouling, etc. [6]. Protective coatings for cultural heritage applications must not only be environmentally and worker-health safe during application but also meet stringent performance criteria. These coatings should be durable, exhibit strong adhesion to the stone substrate, and effectively protect against deterioration agents. In addition, they must achieve this protection without compromising the stone’s water vapor permeability, aesthetic appearance, or the possibility of future retreatments [7]. Costs and limits regarding the application methods also need to be considered.

There is a broad spectrum of choice in the case of protective treatments, with or without a consolidation effect. While hydrophobic coatings might seem to be the most advantageous category, as they prevent the access of liquid water inside the substrate, thus limiting the action of most deterioration mechanisms, they have been used on a large scale in the past with unsuccessful results [8,9]. Various types of waxes and oils, acrylates, polyester and alkyd resins, epoxy resins, silicate esters and alkyl-triethoxysilanes have also been used with different degrees of protective efficacy and durability [6,9]. Recently, there has been increasing interest in the use of nanomaterials as protective coatings, usually as additives in a polymer matrix or in combination with other inorganic compounds. Some well-researched examples are TiO_2_, Ag, ZnO or MgO nanoparticles, with the goal of obtaining super-hydrophobic surfaces [10,11,12,13,14,15].

Nanomaterials can be easily tuned and are an excellent choice for increasing the effectiveness of protective coatings. However, when compared to correctly applied traditional treatments, their high cost and the quantity required may not outweigh the benefits, as was concluded recently in the case of nano-TiO_2_ coatings [16]. Thus, while nanomaterials might offer promising advancements in stone protection, their drawbacks in terms of cost efficiency and potential environmental and health risks must be carefully considered before widespread implementation [17].

Graphene oxide (GO) is a two-dimensional nanomaterial with potential applications as a protective coating in stone cultural heritage, due to its unique structure and surface characteristics. It has some similarities to its “graphene parent” (both are composed of a hexagonal carbon plane), but with varying degrees of structural defects and oxygen functionalities attached. The oxygen atoms are covalently bound to the carbon atoms, converting them from the sp^2^-hybridized state of the graphene planes into the sp^3^-hybridized state with a tetragonal geometry. In comparison with an ideal graphene plane, these oxygen functionalities can be considered as defects, but they offer GO many unique characteristics of high interest for practical applications, a great advantage being its hydrophilic character, due to which stable colloidal solutions might be produced. GO is a nanomaterial with interesting properties, being strong and flexible, with tunable thermal and electrical conductivity, making it a great candidate for a variety of applications [18,19,20]. Large scale production methods are based on the chemical exfoliation and oxidation of graphite flakes, such as the Hummers [21,22] or Marcano–Tour processes [23], which have the advantage of producing larger yields. Subsequently, the obtained GO can be further processed in order to obtain reduced graphene oxide (rGO), a hydrophobic variant, which ideally possesses no oxygen functional groups attached to the carbon atoms, similar to graphene [24].

One of the main advantages of using GO for coatings is the ease of forming continuous films through self-assembly at the liquid–air interface [25,26]. Due to its hydrophilic nature, it is easy to use it as an aqueous suspension and fairly easy to obtain films, which is a great aid in the fabrication of sensors, membranes, or even electronic devices [27,28,29,30]. As a coating material, GO has been successfully used on metal substrates, where it showcased good corrosion inhibition [31,32,33] and better antimicrobial and biocide effects when compared with conventional polymer alternatives, due to the induced oxidative stress [34,35,36,37,38].

Recent studies have begun exploring the possible use of graphene oxide (GO) and reduced graphene oxide (rGO) in the preservation of historical, archaeological, and artistic objects [39,40]. However, there are only a handful of studies discussing the potential use of GO as a coating for stone cultural heritage, which have so far showcased its great effectiveness in protecting the stone against erosion and the lack of leaching in the environment, even after heavy normal or acidic rain simulations [41,42,43,44]. Regarding their influence on the hygric properties of the substrate, GO coatings have so far been tested on concrete, where a reduction by 57% of the capillary water uptake was reported for treated samples with a thick GO layer of 26.2 µg/cm^2^ [45].

To the best of our knowledge, no studies have addressed the changes that a GO coating might induce on the stone substrate characteristics, such as modifying water vapor permeability and capillary absorption (assessed in accordance with EU standard tests), as well as the interaction of GO with the stone substrate or other application methods beside airbrushing.

Of notable interest also is the nanotoxicity and environmental impact of GO, along with mass production and related costs, where current research is still ongoing with no definite conclusions [46,47,48,49,50,51,52,53,54]. Still, the recent improvements in production methods (including more efficient, less costly and greener alternatives), together with the very small quantity usually applied and the evidence of scarce release in the environment [42] indicate that these concerns about using GO for stone cultural heritage applications might not be an impediment in the future.

In this context, we performed a comprehensive study of GO as a protective coating for stone cultural heritage, on two types of stone with a long history of use in Northeast Italy: Vicenza stone and Euganean trachyte. To simulate the impact of weathering on fresh stone samples, we subjected them to an artificial ageing process, according to existing literature [55], and performed all investigations on both natural and aged samples. Water vapor permeability, water capillary absorption, water contact angle, and color change measurements were performed before and after the application of the GO coating, in accordance with existing European Union’s standards for cultural heritage applications. In order to study UV-induced weathering of the GO coating, we then exposed the samples to UV light and performed the tests again. Spectroscopic investigations were also performed to identify the type of interaction of GO with the stone substrates.

Thus, the present study aims to fill the aforementioned gaps by comprehensively evaluating the hygric performance, color impact, and photochemical stability of GO coatings on two stone substrates, both in natural and artificially aged form, with particular emphasis on the effects of UV exposure and coating homogeneity, as well as offering a new perspective on brushing, an application method unexplored so far for GO.

## 2. Materials and Methods

### 2.1. Stone Samples

Stone materials’ properties (compositional, geological origin, textural) affect their bulk behaviour against degradation mechanisms [56,57]. To characterize the efficiency of this protective coating for stone cultural heritage, two types of stone were chosen: the white variation of Vicenza stone (Badia quarry, Berici Hills) and the grey Euganean trachyte stone (Montemerlo quarry, Euganean Hills), due to their different composition and geological origin, as well as textural features. Both types of stone are extracted from active quarries in Northeast Italy and have been employed since antiquity as dimension stones or in the construction of sculptural complexes.

Vicenza stone is an organo-genic limestone that is distinguished by a high level of porosity (25–29%) and the inclusion of micro- and macro-clastics of foraminifera, bryozoans, algae, and echinoderm [57,58,59]. It has an exceedingly heterogeneous structure due to its composition, and is comprised mostly of calcium carbonate (above 90%) with trace amounts of silicon, aluminium, and iron oxides. Due to its high specific surface dictated by its large porosity, the characteristic weathering forms include dissolution and pulverization, which are commonly accompanied by biological colonization [59].

Euganean trachyte encompasses several distinct varieties of rock extracted from the Euganean Hills. These varieties share a characteristic porphyritic texture and a predominantly gray color, which can occasionally range to brown or yellow hues. [60,61]. Although they might belong to the same quarry basin, these stones exhibit a relatively wide array of mechanical performance, strongly depending on their pore characteristics; their porosity ranges from 10–19% [60]. Montemerlo trachyte is extracted from one of the last still active quarries and is considered a type of igneous rock, with a characteristic fine-grained matrix. Its chemical composition consists of a large amount of SiO_2_ (around 60–65%), with important contributions from Al_2_O_3_ (around 17%), alkali oxides (Na_2_O and K_2_O, about 5% each) and lower amounts of Fe_2_O_3_, MgO, and CaO, highlighting its large degree of heterogeneity [61]. Having excellent mechanical properties, its degradation mechanisms are generally dependent on pollutant exposure, resulting in distinctive decay traits, such as contour scaling, flaking, and exfoliation, which leads to granular disintegration and crumbling [62]. In the presence of SO_2_, black crust forms are also common, which are prone to separation and structural damage [63].

Stone samples were cut according to the test to be performed and part of them subjected to artificial weathering (see methods below). Cubic samples (4 × 4 × 4 cm) were used for water capillary absorption tests (6 samples, 3 for natural stone, 3 for aged stone), for contact angle measurements and colorimetric tests (3 samples) and Raman (single stone specimen of each type: uncoated natural/aged stones, GO coated natural/aged stones, UV-exposed GO coated natural/aged stones). Disks (5 cm diameter, 1 cm thickness) were used for water vapor permeability tests (6 samples, 3 for natural stone, 3 for aged stone).

### 2.2. Graphene Oxide Synthesis and Application

#### 2.2.1. Synthesis

For our study, an in-house sono-chemical exfoliation method using the precursors of the Marcano–Tour process was used for GO synthesis, which included additional steps of ultrasonication, washing, centrifugation and decantation, and was previously used to produce GO with exceptional self-assembly properties [23,26,64]. Briefly, 270 mL of H_2_SO_4_ (97%, Nordic Invest SRL, Cluj-Napoca, Romania) and 30 mL of H_3_PO_4_ (85%, Sigma Aldrich, St. Louis, MO, USA) were mixed in a 9:1 volume ratio. After 5 min, 2.7 g of graphite (99.9995%, powder, 100 mesh diameter, Alfa Aesar, Haverhill, MA, USA) was added in small portions for 20 min (i.e., a fifth of the total quantity at a time, with 3 min between additions) under continuous mixture in an ice bath. Then, 12 g of KMnO_4_ (99%, Sigma Aldrich, St. Louis, MO, USA) were added in small portions for 15 min, while maintaining a continuous mixture and the ice bath. The initial mixture was then left for 60 min under stirring in the ice bath. Then, the ice bath was removed, and the mixture was left for 48 h at room temperature in ambient atmosphere. The second step of the synthesis method consisted of slowly adding 200 mL H_2_O_2_ (3%, precooled, Hipocrate 2000 SRL, Bucharest, Romania) for 20 min to the mixture, which was stirred in an ice bath. Then, the mixture is left for another 20 min to cool in the ice bath while stirring. Afterwards, the mixture was centrifuged for 10 min at 6000 rpm, and the supernatant was decanted away. Then, the remaining solid underwent successive washings with 200 mL H_2_O MQ, 100 mL HCl (35%, Sigma Aldrich, St. Louis, MO, USA), and 100 mL absolute ethanol (SC Nordic Invest SRL, Cluj-Napoca, Romania), in that order. After each washing, the mixture was subjected to a series of 15 min of ultrasonication and 10 min centrifugation at 6000 rpm. The supernatant was decanted away each time. The washings with HCl and absolute ethanol were carried out twice. After the last decantation, the remaining solid was mixed with 400 mL H_2_O MQ, ultrasonicated for 60 min, and kept in a sealed jar for 4 days. The final step of the synthesis consisted in harvesting with a syringe the superior fraction from the jar and represented the final product of the synthesis. The inferior fraction (about one fifth of the total amount) consisted of a more viscous GO suspension, which may contain unexfoliated graphite, and was not used. Size and charge distributions, as well as other physico-chemical parameters of GO dispersions synthesized through this method, have been thoroughly investigated elsewhere [26,64].

#### 2.2.2. Application

Given the critical importance of aesthetic compatibility in conservation treatments, preliminary brushing trials were conducted on stone samples before applying the GO suspensions. These experiments aimed to determine the optimal combination of GO concentration and number of applications. In the end, a GO water dispersion with a concentration of 0.5 mg/mL and 2 applications by brush were the limits for which a color change was not immediately perceivable, and we decided to use this for further experiments, as a compromise ensuring adequate coating deposition. Spray application was also briefly tested but, at this concentration, a more homogeneous coverage was obtained by brushing. The two brush applications were performed immediately one after the other and the stone samples were rotated 180 degrees between applications to improve the homogeneity of the coating.

### 2.3. Characterisation Methods

XRD and FT–IR spectroscopy were used to characterize the stone substrates, to confirm the structure and chemistry of the synthesized GO and to evaluate possible changes due to UV exposure. Raman spectroscopy was used in order to investigate changes in the oxidation degree of the GO coating following the application on the stone surface and the subsequent UV exposure.

A Renishaw InVia Reflex Raman spectrometer with an air-cooled RenCam CCD detector was used to capture the Raman spectra of dried GO films and GO-coated stone samples. The excitation source used was a 785 nm laser line, with a power of 106 mW. A 10 s exposure duration at 10% power was used. In all cases, the spectral resolution was 4 cm^−1^.

Transmission FT–IR experiments on dried GO films were performed using a Thermo Fisher iS 10 Nicolet FTIR spectrometer using the KBr pellet method (1:100 dilution ratio). For each spectrum, 128 scans were acquired in the 400–4000 cm^−1^ range, with a spectral resolution of 4 cm^−1^. FT–IR characterization of the stone samples was performed using a JASCO 6600 FT–IR spectrometer in transmission mode within the range of 400–4000 cm^−1^, at room temperature, with a spectral resolution of 4 cm^−1^, using the KBr pellet technique (1:100 dilution ratio). The structural characterization of the stone samples and dried GO films was determined via X-ray Diffraction (XRD) performed by means of a Panalytical Empyrean diffractometer equipped with a Cu X-ray tube (λ = 1.5406 Å) and a PIXcel^3D^ two-dimensional detector with a 256 × 256 array sensor, operating in a Bragg–Brentano configuration at 40 kV and 40 mA.

To assess the effects of the protective coatings on weathered stones, we subjected a part of the stone samples to an artificial thermal ageing process, which consisted in heating the dried samples to 400 °C for one hour [55]. In order to assess the effect of the thermal treatment on the open porosity of the stone samples, we performed Mercury Intrusion Porosimetry (MIP) investigations using a Thermo Fisher PASCAL porosimeter with a double module—Pascal 140 and Pascal 240—which operated at pressures of 40 MPa and 200 MPa. Pressure measurement (which is used to calculate the pore distribution) has an accuracy of 0.2–0.25% and has a lower limit of 0.0074 um for pore radius. This allowed the effective measurement of pores with a diameter between 3.7 nm and 150 µm. The two measurements were performed, combined, and processed through the SOLID version 1.4.1 software. The liquid–solid contact angle for the mercury-stone system was 141.3°. For both Vicenza stone and Euganean trachyte, a sample each of the natural and aged stone were evaluated.

To verify the initial effect of UV exposure on the fresh GO coating, we exposed the treated stone surfaces to a UVC lamp (Helios Italquartz low-pressure Hg lamp) with a total power of 25 W. The lamp power at wavelength λ = 254 nm was 7.5 W and was placed at 1 cm from the treated surface of the samples. The exposure time was set to 7 h for all samples.

To investigate the stone surface morphology with and without the GO coating applied, we used an Olympus SZX12 microscope coupled with a digital camera for optical microscopy pictures, and the FEI Quanta 200F for SEM micrographs. Secondary electrons were collected using an E-T detector (10 kV, working distance of 9–10 mm, spot size 3.0–3.5) to obtain the SEM micrographs.

Water vapor permeability (WVP) tests were performed in accordance with the EN 15803:2010 standard for cultural heritage applications in wet cup conditions [65]. Three disks (5 cm diameter, 1 cm thickness) of each type of stone previously preconditioned, were used to cap a cup containing a saturated solution of KNO_3_ with the goal of maintaining an internal relative humidity of 93%. The cups were sealed with Parafilm tape and then left in a desiccator at a temperature of 23 °C and a relative humidity of 50%. Measurements of the cup weight, desiccator temperature, relative humidity, and air pressure were made every 24 h, until the system reached a steady state.

Water capillary absorption (WCA) tests were performed according to the EN 15801:2009 standard for cultural heritage applications [66]. For each type of stone, three cubic samples (4 × 4 × 4 cm), after preconditioning, were weighed before the experiment, and then placed in a container with the treated face on top of a 5 mm layer of filter paper kept soaked with MQ water. The experiment was performed at 23 °C. The samples were weighed at regular times after the start of the experiment, until the mass change between two consecutive measurements did not exceed 1%.

Water contact angle tests were performed in accordance with EN 15802:2009 standard, used in cultural heritage applications [67]. Measurements were performed on the same treated face of the three cubic samples used for each type of stone on the WCA tests. For the experimental setup, a Dino-Lite microscope was used to photograph the 10 µL water drops. For each sample, 15 measurements were made by photographing the drops 10 s after their contact with the surface. The photographs were then analysed to determine the contact angle, through the DinoCapture 2.0 software, and the results were averaged.

For the colorimetry tests, a CM-2600d Konica Minolta spectrophotometer was used to record the reflectance spectra of each sample, collected in the range of 360–740 nm, with a 10 nm resolution. The measurements were performed in the CIEL*a*b* color space, where L* is the lightness and a* and b* are the chromaticity coordinates in the red-green and the yellow-blue direction, respectively. For each point analysed, three measurements were averaged, and were performed in the SCI (specular component included) mode, on the same cubic stone samples also used for the WCA and contact angle measurements. Using a mask, the same five points on the treated face of the samples were analysed. In accordance to the existing literature, a color variation ΔE* greater than 3 becomes visible, while a variation greater than 5 is considered unacceptable for cultural heritage [68].

Water contact angle (WCA) and water vapor permeability (WVP) measurements were conducted on the same stone samples in the following sequence: for both natural and aged stones, three cubes/discs were tested—first in the uncoated state, then after applying the GO coating, and finally after exposing the GO coating to UV light. Contact angle and colorimetric evaluations were carried out on three cubic samples (with measurements taken on the same face) at four stages: on the natural stone, after ageing, following GO coating, and after subsequent UV exposure. FT–IR and XRD analyses were performed once for each sample type (using GO on glass slides both before and after UV exposure, as well as on natural and aged stone samples). Additionally, Raman spectra were obtained from a single specimen for each condition: uncoated natural/aged stones, GO-coated natural/aged stones, and UV-exposed GO-coated natural/aged stones.

## 3. Results and Discussion

### 3.1. Characterization of Stone Substrate Specimens

**Figure 1:**
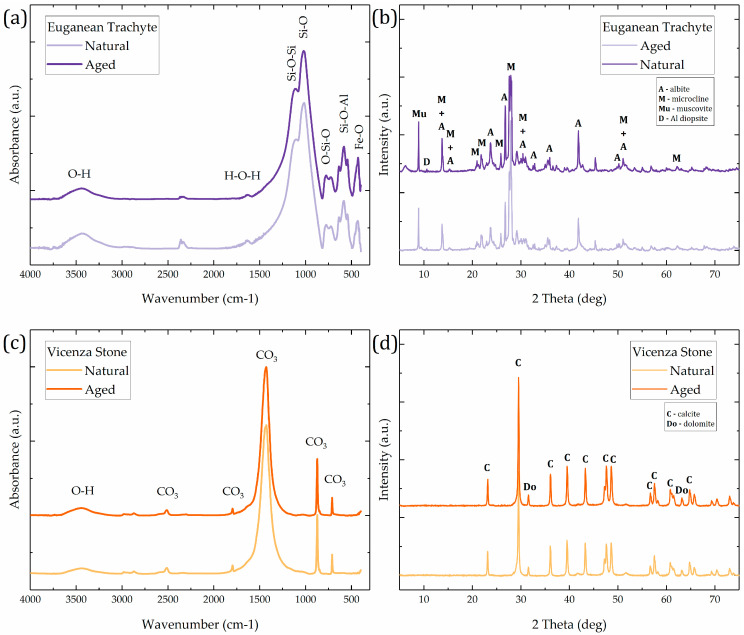
FT–IR absorption spectra and XRD diffractograms of Euganean trachyte (**a**,**b**) and Vicenza stone (**c**,**d**) samples. In the FT–IR spectra, the weak absorption at about 3400 cm^−1^ and 1630 cm^−1^ is ascribed to a small amount of moisture in the samples or in the KBr powder used.

In conservation practice, protective coatings are typically applied to aged and weathered stone. Therefore, both Euganean trachyte and Vicenza stone samples were analyzed before and after undergoing artificial aging. This approach allowed for a comparison of GO coating performance on both freshly quarried and artificially aged stone substrates. Transmission FT–IR, using the KBr pellet method, and XRD analyses were performed on powdered stone samples (Figure 1).

The following absorption bands of the main minerals in Euganean trachyte can be identified in the FT–IR spectrum (Figure 1a): δ(Si-O-Si) at 1115 cm^−1^, ν(Si-O) at 1015 cm^−1^, δ(O-Si-O) at 781 cm^−1^, δ(Si-O-Al) at 593 cm^−1^, and ν(Fe-O) at 430 cm^−1^. In both FTIR and XRD, no remarkable differences between natural and aged trachyte sample can be observed (Figure 1a,b). In particular, the reflection ascribed to Albite (A), Microcline (M), Muscovite (Mu), and Aluminium Diopside (D) [60,69] persist unchanged and well recognizable.

The FT–IR absorption spectrum of Vicenza stone (Figure 1c) exhibits typical Ca(CO)_3_ absorptions: the ν_4_ (at 712 cm^−1^), ν_2_ (at 873 cm^−1^), ν_3_ (at 1440 cm^−1^), and combination overtones of ν_1_ + ν_4_ (at 1799 cm^−1^) and 2ν_2_ + ν_4_ (at 2515 cm^−1^) of the CO_3_^2−^ ion [70]. XRD patterns of the two Vicenza stone samples highlight the calcite (C) and dolomite (Do) reflections [70]. As observed in Euganean trachyte, FT–IR and XRD did not highlight any differences between the natural and aged Vicenza stone samples (Figure 1c,d).

**Figure 2:**
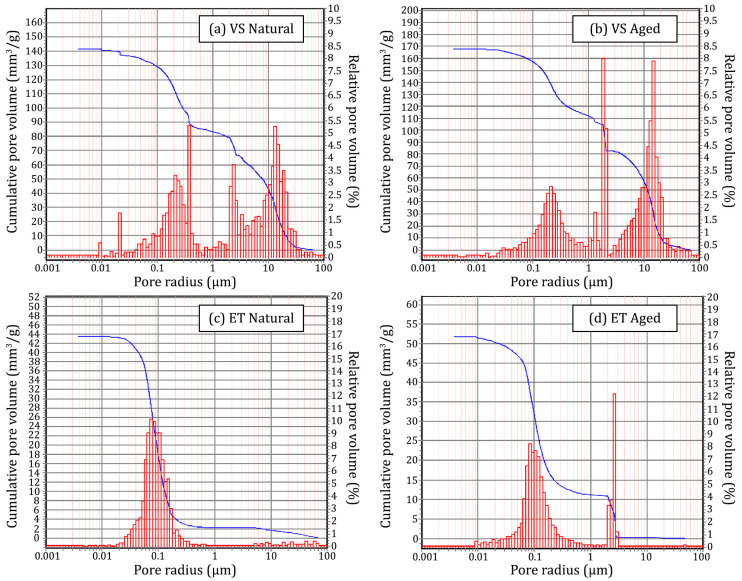
Cumulative pore volume (blue) and relative pore volume distributions (red) of natural and aged Vicenza stone (**a**,**b**) and Euganean trachyte (**c**,**d**).

Through MIP tests, we analysed the porosity of natural and aged stone samples. Pore size distributions can be observed in Figure 2, while the stone densities and porosity-related parameters are presented in Table 1. In the case of Euganean trachyte, we first see a unimodal distribution with a peak at about 0.08 µm, which changes to a bimodal distribution following the aging process. A new peak appears at about 2.5 µm, as the thermal treatment damages the matrix and connects existing pores. For Vicenza stone, the ageing process enhances the relative amounts of 1 µm and 10.5 µm pores. Overall, the ageing process does not change the general shape of the pore distributions, but rather generates pores of specific sizes as a result of thermal stress, which collapses pores in the stone matrix [55].

These results agree with the literature: Euganean trachyte represents a compact silicate stone substrate with medium open porosity and numerous impurities of Al, Na, K, Fe, and Mg present in the matrix [60], while Vicenza stone samples offer a highly porous carbonate stone substrate with large open porosity [58], but a rather homogeneous chemical composition. After thermal treatment, an increase of the open porosity and a different pore size distribution was obtained, confirming its efficacy as an artificial ageing process [55].

### 3.2. Preliminary Experiments and Analysis of GO Coatings on Glass Slides

**Figure 3:**
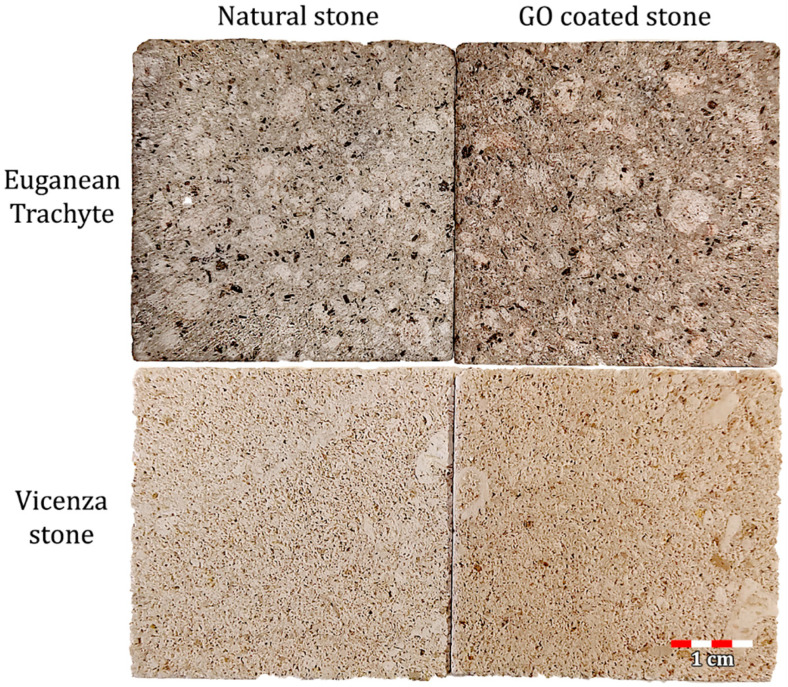
Digital images of natural and GO coated Euganean trachyte and Vicenza stone samples.

After the preliminary trials, 2 applications by brush of a GO water dispersion with a concentration of 0.5 mg/mL were chosen. While initially imperceptible, a slight color change became visible over time (Figure 3). Despite this, we decided to use this for further experiments as a compromise between maintaining aesthetic compatibility and ensuring a sufficient amount of GO on the stone surface for reliable results in subsequent experiments.

Photochemical stability is also an important requirement for protective coating. Since it is known that GO can undergo reduction by action of light [71], and to evaluate how the increase in hydrophobicity due to reduction could actually modify GO coating properties, the immediate effect (up to 7 h) of UV exposure on the fresh GO coating was studied. After some preliminary trials, a 7-h exposure time was selected because no additional changes were visibly noticed in the color of the coating or in the XRD diffractograms of the UV exposed GO coatings after that time.

**Figure 4:**
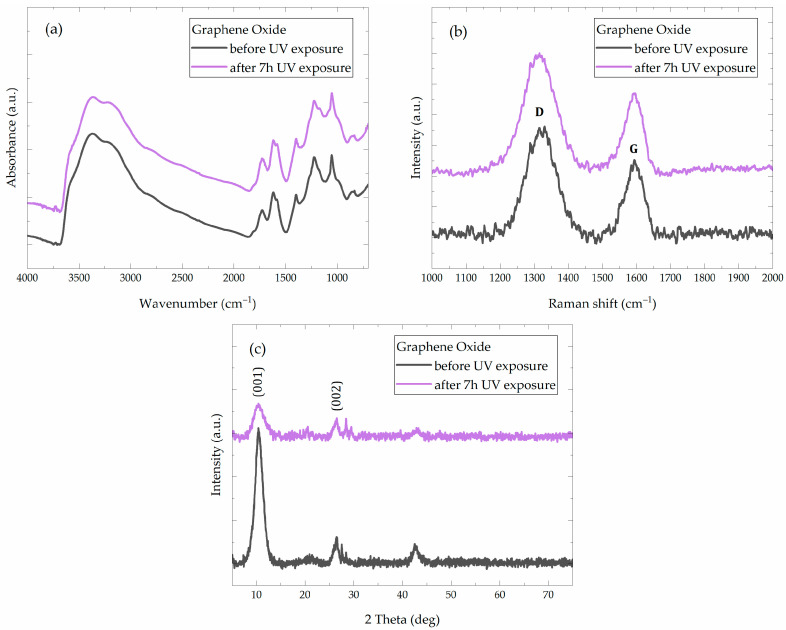
Analysis of dried GO films before and after their exposure for 7 h to UV light (λ = 254 nm): (**a**) Transmission FT–IR spectra (KBr pellet method), (**b**) Raman spectra (785 nm excitation wavelength), (**c**) XRD diffractograms.

In order to characterize the nanomaterial when applied, we also prepared GO coatings on glass slides, using the same 0.5 mg/mL GO water dispersion used for the stone brushing, which were left to dry in ambient conditions for a week. FT–IR, Raman spectroscopy, and XRD analyses were performed on regular and UV-exposed coatings (Figure 4).

Transmission FT–IR spectra of the obtained films can be seen in Figure 4a. The characteristic GO absorption bands can be observed, in accordance with the existing literature [72,73,74,75]. Thus, the wide absorption band at about 3420 cm^−1^ appears due to the ν(O-H), the band around 1735 cm^−1^ is given by the ν(C=O), the band around 1640 cm^−1^ is attributed to the ν(C=C) bonds, and the absorption band around 1430 cm^−1^ is assigned to ν(S=O), which corresponds to leftover sulphate from the synthesis process. Moreover, in the 1250–1000 cm^−1^ region the absorptions due to ν(C-OH), ν(C-O-C) and, ν(C-O) were observed at about 1220, 1165, and 1050 cm^−1^, respectively.

The Raman spectra (Figure 4b) showcase the characteristic D (at 1320 and 1315 cm^−1^ for GO and GO–UV, respectively) and G bands (at 1594 and 1591 cm^−1^ for GO and GO–UV, respectively) of graphene materials [64,75,76]. The D band is associated with the breathing mode of carbon rings and is activated by the presence of structural defects or disorder (sp^3^ carbon), such as oxidized regions or edge effects, while the G band corresponds to the in-plane stretching vibrations of sp^2^ carbon-carbon bonds in the graphitic lattice. The intensity ratio of these bands (I_D_/I_G_) provides information on the degree of disorder and the density of defects in GO, reflecting its oxidation level. Through fitting the two bands, the calculated I_D_/I_G_ ratios were 1.47 and 1.48 for the GO and GO–UV samples, which corresponds to a highly oxidized structure.

While only minor changes appear in the FT–IR or in the Raman spectra of GO samples, a noticeable decrease in crystallinity is observed for the XRD diffractograms (Figure 4c). The characteristic (001) peak is found at 2θ values of 10.51° for both GO and GO–UV and corresponds to an interlayer distance of 8.4 Å in both cases, in accordance with Bragg’s law. The intensity of this reflection is greatly reduced following the UV exposure, which suggests a decrease in crystallinity. A lower intensity peak can also be seen in all samples at 2θ values of about 26.46° for GO and 26.39° for GO–UV, which is characteristic to (002) reflections of leftover graphitic domains [72]. We can also see an additional peak at 2θ values of 42.69° and 42.99° for GO and GO–UV, which corresponds to the short-range ordering in the stacked carbon layers, which indicates the presence of semicrystalline turbostratic graphitic domains [77,78,79].

Although only slight changes appear in the FT–IR and Raman spectra, the UV exposure greatly changes the color of the samples prepared on glass slides. The films become darker, which might be an indication of the expected photoreduction of the oxygen functional groups (i.e., GO is partially converted to the so-called reduced graphene oxide, rGO) [71]. Together with the observed changes in the XRD patterns, these findings suggest that the UV exposure induces significant surface modifications and a decrease in crystallinity, while the bulk structure of the GO coating remains mostly unchanged.

### 3.3. Application of GO Suspensions on Stone Substrates

#### 3.3.1. Surface Analysis of GO Coatings on Stone

After 2 applications by brush of the 0.5 mg/mL water suspension, the GO quantity applied on the stone surface was calculated to be about 1.96 µg/cm^2^.

**Figure 5:**
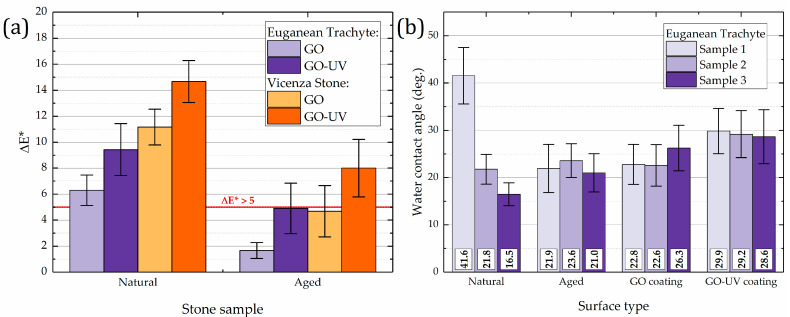
(**a**) Color variations calculated for each coating on natural and aged Euganean trachyte and Vicenza stone samples. (**b**) Averaged water contact angle measurements for three samples of Euganean trachyte. For each sample, 15 measurements were made.

Colorimetric investigations were performed by making three measurements on the same five points on the surface of each sample, by using a mask. Color variation ΔE* was calculated, and the results are represented in Figure 5a.

Applying GO coatings on natural, fresh stone samples leads to a color change greater than the accepted value for cultural heritage applications. Moreover, it seems that the UV weathering of the coating further increases the color difference. For a better picture of the color change, a HEX color chart of all colorimetric measurements carried out on the same five points on the treated surfaces can also be seen in Figure S1 (Supplementary Material). Table S1 (Supplementary Material) shows the calculated variation of L*, a*, and b*, as well as ΔE* for each sample. For all samples, the largest change induced by the GO or GO–UV coatings appears in the lightness component (L*), which corresponds to a darkening of the perceived color. In each case, the weathering process induces a further reduction of L*.

Applying GO coatings to aged stone samples (intended to mimic the effects of natural weathering on monuments) resulted in a smaller overall color change compared to coatings applied to fresh stone. In some cases, the change remained within acceptable limits. This is especially true for Euganean trachyte, which is a grey type of stone, for which a reduction of L* is harder to perceive. In the case of the white Vicenza stone, with a higher L* component, the application of a GO coating and its exposure to UV radiation would lead to a much larger color difference than the acceptable range.

Water contact angle measurements were performed on the same stone samples used for colorimetry, to investigate the degree of hydrophilicity or hydrophobicity due to the presence of the GO coatings. Tests were performed on three samples of natural stone, which were subsequently aged, coated with GO, and later exposed to UV light. In each case, no reliable measurement was possible for the Vicenza stone samples, due to their high-water absorption, a consequence of their large open porosity. Results were only obtained for Euganean trachyte stone samples and are represented in Figure 5b as the individual values for each of the three stone samples used. A large variation in water contact angle values is naturally observed for the results obtained from the fresh stone samples, which is not observed after the artificial ageing process takes place. Most notably, however, is that the GO coating does not change the hydrophilicity of the stone surface. A very slight shift towards hydrophobicity is noticed after the UV exposure, which may account as a confirmation of the photoreduction of GO when subjected to UV radiation.

**Figure 6:**
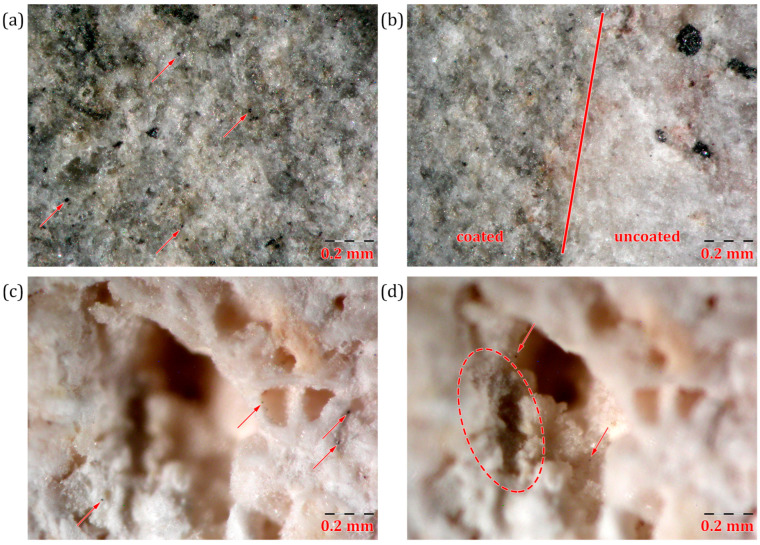
Optical images of: (**a**) Euganean trachyte coated with GO; (**b**) edge of coated area on the surface of Euganean trachyte; (**c**,**d**) Vicenza stone coated with GO, with a focus on the exterior and interior of a pore. Red markings indicate distinctive features observed.

In order to elucidate the interaction mechanisms of the GO coatings with the stone substrate, we performed additional investigations on the morphology of the coatings. Figure 6 shows different features observed through optical microscopy after the application of a GO coating.

Optical microscopy revealed the presence of black flakes on the coated surfaces. These flakes may be GO agglomerations that were either present in the original dispersion or formed immediately after application. The observed agglomerations could also be a result of GO’s known self-assembly behavior at liquid interfaces, which could have occurred immediately after brushing. Figure 6b shows a clear demarcation line between the coated and uncoated areas of the surface. Although dark agglomerations are noticed on the coated section, there is also a visible overall darkening of the surface. Figure 6c,d shows the image of a 1 mm wide pore on a GO coated Vicenza stone. A dark area can be identified inside the pore, where possible GO agglomeration might have occurred. Generally, a relatively homogenous darkening effect is shown on coated areas, with scarce but observable individual GO agglomerations.

**Figure 7:**
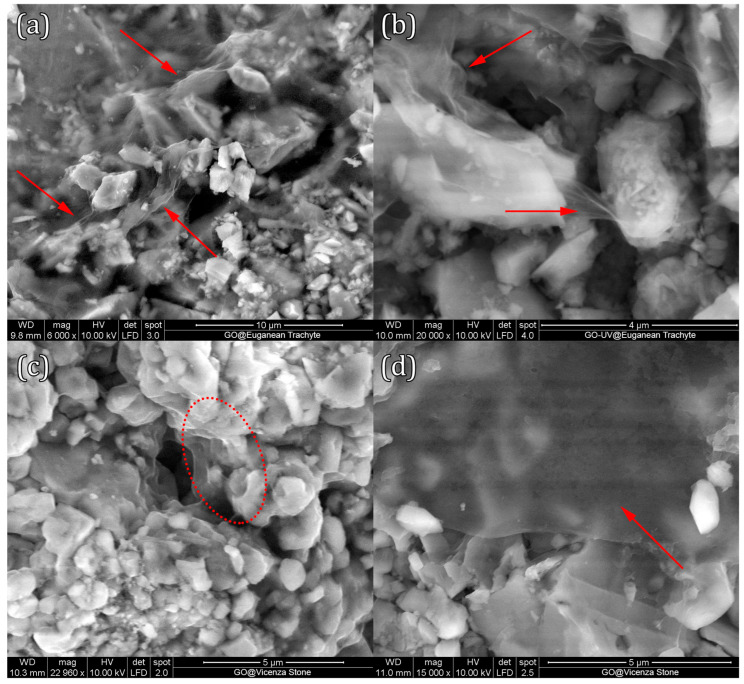
Selected SEM micrographs of GO/GO–UV coated Euganean trachyte (**a**,**b**) and Vicenza stone (**c**,**d**) samples. Red markings indicate GO coverage of the stone substrate.

SEM investigations were performed on stone samples, before and after application of GO coatings and after UV exposure. Selected images showcasing the most interesting features observed are shown in Figure 7. In general, an inhomogeneous distribution of the GO or GO–UV coating is observed, varying from thin and transparent veils covering surface and pores, to thicker agglomerated spots of different dimensions and shapes, particularly found in pores and edges. No observable differences can be seen in the SEM micrographs between the aspect of GO and GO–UV coatings.

**Figure 8:**
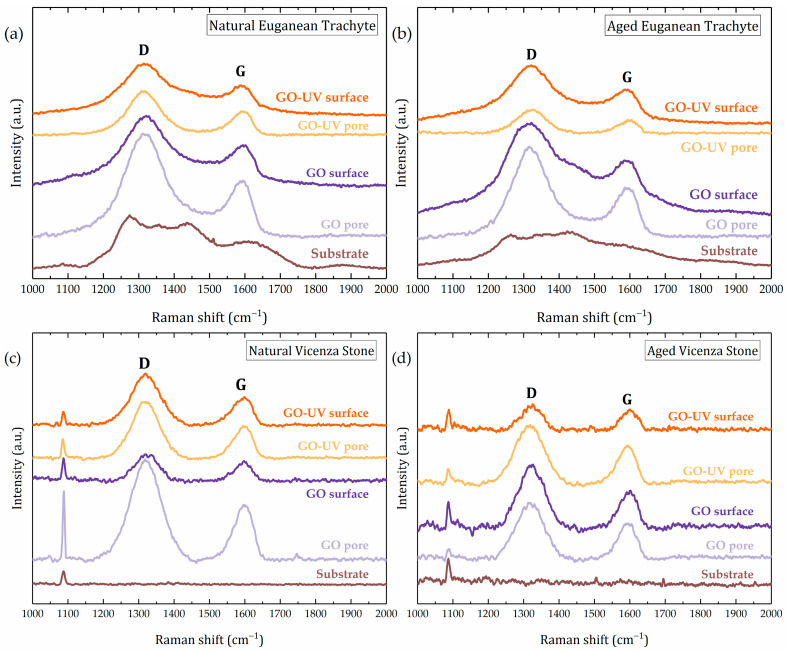
Raman spectra of GO and GO–UV coated natural (**a**,**c**) and aged (**b**,**d**) Euganean trachyte and Vicenza stone samples, respectively. Spectra have been taken with the aid of the microscope from visible flat surfaces or pores.

Consequently, we tried to obtain further information from the Raman spectra of pore and surface domains with the aid of a micro-Raman system, with a spot size of about 2 µm (Figure 8). In the case of Vicenza stone samples, the characteristic ν_1_ symmetric stretching of CO_3_ ion is observed at 1090 cm^−1^ [70]. Each spectrum was analysed, and the I_D_/I_G_ ratio was evaluated. By fitting the two D and G bands, the full width at half maximum (FWHM) of each band was recorded. The results are shown in Table 2.

A noticeable trend is visible when comparing the D and G band parameters of GO and GO–UV coatings between pore and surface domains for Vicenza stone. It seems that UV exposure is affecting the surface domains more than pore domains, for which almost no change was noticed in the I_D_/I_G_ ratio. Looking at FWHM of D and G bands of pore domains, it seems that the FWHM values of both bands are closer to the values obtained for reference GO and GO–UV samples. Larger variations appear for surface domains, especially for the D band.

Based on these results, it can be supposed that UV exposure is only able to reduce the first few atomic layers of GO, hence the color change, but in-depth alterations might not occur due to the low penetration depth of the UV light. Concerning Euganean trachyte samples, trends are harder to observe, mostly due to bands corresponding to some Raman active mineral phases or some organic contamination, which underlies the D and G bands of GO.

Differences in FWHM of both D and G bands have been identified, which is an indicator of the interaction with the stone substrate. We attempted to identify the type of bond formed through ATR FT–IR investigations but, due to the very low amount of GO (about 1.96 µg/cm^2^), no signal originating from the coating was observed.

To our knowledge, physical abrasion has not been analyzed so far on GO stone coatings. However, recent leachate analysis of GO-coated stone has demonstrated that GO does not leach into the environment after substantial water exposure on coated dolomite surfaces [42]. This observation supports the hypothesis that ions released from the stone (Ca^2+^, Mg^2+^, Al^3+^) due to the acidity of GO can act as chemical crosslinkers between GO sheets, creating a strong bond with the stone substrate. [41,80,81]. This also eliminates some of the concerns related to the associated health or environmental risks after the coating is applied. Nonetheless, further surface investigations should be performed to clearly identify the type of interaction between GO and the stone substrate.

#### 3.3.2. Evaluation of Hygric Properties

**Figure 9:**
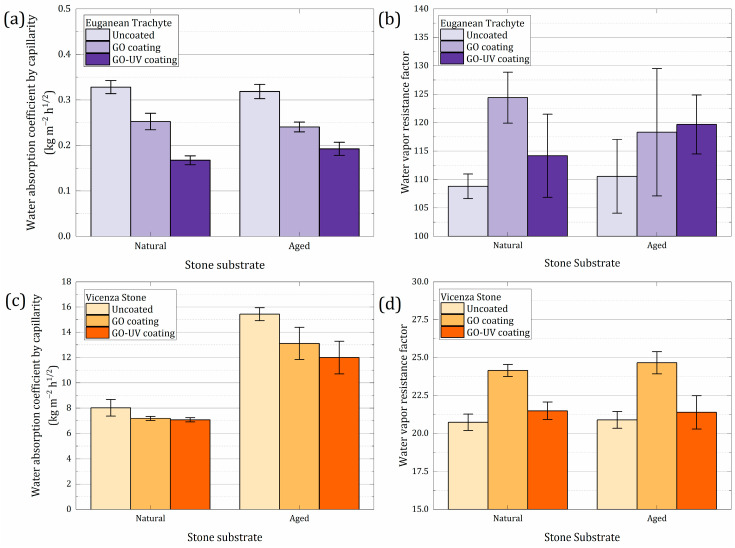
Water absorption coefficient by capillarity and water vapor resistance factor of Euganean Trachyte (**a**,**b**) and Vicenza stone (**c**,**d**) samples.

Sound and aged Euganean trachyte and Vicenza stone samples were used for the analysis of GO coating influence on the water vapor resistance factor and the coefficient of absorption through capillarity. These properties are essential to evaluate the performance of a coating on the most relevant moisture transport mechanisms in stone. Three samples were used for each type of stone. First, the uncoated samples were tested, and the tests were carried out again after coating the same samples and later exposing them to UV light. Results are represented in Table 3 and a graphical version is pictured in Figure 9.

The artificial aging process did not significantly alter the water-related petrophysical properties of the stone samples, with one exception: the capillary water absorption coefficient of Vicenza stone. This property showed substantial differences after aging, likely due to changes in pore size distribution, which, as confirmed by MIP measurements, greatly increased the open porosity of this stone type. GO–UV coatings generally outperformed fresh GO coatings, likely due to UV-induced surface reduction, which imparted a slight degree of hydrophobicity. For coated samples, a reduction in the water capillary absorption coefficient is observed, especially for Euganean trachyte stone samples, where a maximum decrease of 49.0% was reported for sample coated with GO–UV (Table 3). Although lower, a significant effectiveness is still seen for a stone substrate with a large water capillary absorption coefficient such as Vicenza stone, where a decrease of up to 22.3% is reported when the sample was coated with GO–UV (Table 3).

The quantity of absorbed water per unit area (Kg/m^2^) in relation to time (s^1/2^) can be seen in Figure S2 of the Supplementary Material. For Vicenza Stone, most notably, artificial ageing leads to a quicker absorption, which is slowed down by the coating.

Differences between GO and GO–UV coatings are apparent only after approximately 30 s^1/2^, though both eventually reach the same water saturation plateau. In contrast, Euganean trachyte exhibits a different behavior: GO coating reduces both water absorption rate and total absorption, preventing it from reaching the saturation plateau. These effects are amplified in GO–UV due to increased hydrophobicity.

Fresh GO coatings altered water vapor resistance by up to 14.3% in Euganean trachyte and 18.0% in Vicenza stone. UV exposure significantly reduced this effect, making the change indistinguishable from the natural variability of the stone itself. Minimal alteration of water vapor diffusion is a crucial requirement for stone protective coatings. Despite the uneven distribution, GO and GO–UV coatings show an excellent ability of reducing the water absorption, while maintaining a good vapor permeability. Based on Raman results for the Vicenza stone, we can theorize that the exposure to UV radiation is strongly affecting the thin surface GO domains, rather than the pore GO domains, where thicker agglomerations were noticed. Due to geometric considerations and their different thickness, pore and surface domains suffer different levels of photoreduction.

The excellent water vapor permeation of GO, compared to its reduced counterpart, [81,82,83] suggests that UV radiation primarily impacts the coating’s surface domains. This partial photoreduction likely reduces water capillary absorption by hindering water molecule access to the pores. However, the pore domains remain largely unaffected, allowing water vapor to permeate through the stone substrate.

A different explanation can relate to the intrinsic barrier nature of the GO, which can selectively allow the permeation of water depending on the interlayer distance [82]. Water transport through GO membranes occurs via low-friction flow through nanocapillaries formed by pristine graphene empty spaces. Oxidized functional groups increase interlayer spacing, significantly influencing water permeation. Conversely, reduced graphene oxide, with its smaller interlayer distance, exhibits reduced water permeability. Modifications to oxygen functional groups or the presence of cations, particularly their properties (e.g., electronegativity, ionic charge, hydration radius), can further modulate water permeation. In our case, partial reduction and interaction with stone-released cations could have contributed to regulating interlayer water transport [80,81].

Future work should ideally fully elucidate the interaction between GO and the stone substrate, as well as analyzing a larger variety of substrates. Moreover, additional application methods should be experimented. We believe that the influence of the application method of choice can also lead to dramatic differences between the observed behaviour and performance of the GO coating. While we report here micrometer-sized GO agglomerations, there has not been any mention of this behaviour so far in other publications on GO coatings for stone cultural heritage, where airbrushing was used for its application. It might be that brushing, a very simple and facile method, widely used in restoration practice, generates the proper conditions for the self-assembling of GO during the subsequent drying of the excess solvent, while airbrushing and its inherent mechanism hinders it. Tailoring the GO synthesis method, the dispersion concentration and the number of applications can also potentially improve the homogeneity and reduce the darkening effect.

In practical applications, GO coatings could be directly applied to historical monuments. Natural UV exposure will inevitably induce photoreduction, further enhancing water resistance but darkening the coating, highlighting the need for future studies to determine the optimal concentration to be applied and perform suitable durability tests.

Functionalizing GO may help prevent agglomeration, as has recently been demonstrated for both graphene and graphene oxide [84,85,86]. Additionally, incorporating lighter nanomaterials (such as ZnO or TiO_2_) into GO composites could mitigate color variations, a key limitation in using GO as a protective coating for stone cultural heritage. Notably, a recent study opened a new direction of research on this subject by depositing ZnO nanorods onto graphene nanoplates [87]. The resulting composites, when airbrushed onto substrates, such as Noto stone, Carrara marble, and common yellow brick, exhibited strong antibacterial properties without inducing significant color changes (ΔE* < 5)

## 4. Conclusions

A systematic analysis of the performance of GO as a protective coating was performed for two types of stone, Euganean trachyte and Vicenza stone, which have been historically used as dimension stones for buildings or pavements in Northeast Italy. An artificial ageing process was also used for the stone samples to simulate the conditions of real monuments. Coatings were obtained by twice brushing a 0.5 mg/mL GO water suspension, resulting in a coverage of about 1.96 µg/cm^2^. To investigate the stability of the coatings against UV radiation, a 7-h exposure at λ = 254 nm was also performed.

Meeting the specific requirements for cultural heritage protective coatings, GO coatings effectively reduced water absorption by capillarity by an average of up to 49.0% for Euganean trachyte and 22.3% for Vicenza stone, after 7 h of UV exposure, while maintaining minimal change in water vapor permeability.

Colorimetric analysis revealed better results for coatings on artificially weathered stones. No coating on natural stone met the ΔE* < 5 limit, regardless of UV exposure. On artificially aged Euganean trachyte, color changes remained within this limit, even after UV exposure. In the case of the artificially aged but lighter Vicenza stone, the UV exposure leads to a color variation above the acceptable threshold. While color change perception depends on the original stone color, limiting its applicability, GO coatings may still be suitable for specific cases (e.g., gray stones, concrete).

UV-induced photoreduction plays a dual role in this system. On the one hand, it results in a noticeable and permanent darkening of the GO coating. On the other hand, photoreduction leads to chemical alterations that slightly enhance the hydrophobicity of the coating, thereby contributing to the observed decrease in capillary water absorption. Raman spectroscopy results support the interpretation that UV exposure primarily affects the thin surface layers of the GO coating, while the thicker pore-associated domains remain less altered. This differential variation suggests that the protective performance of GO may arise from a combination of its intrinsic barrier properties and selective photoreduction effects.

Our study confirms that GO, particularly after UV-induced photoreduction, can significantly reduce water absorption in stone substrates while preserving essential water vapor permeability. Although issues such as coating homogeneity, color alteration, and long-term durability remain to be fully addressed, the promising results obtained here lay the groundwork for further investigations. Future research should focus on optimizing application techniques, exploring functionalized or composite formulations, and conducting trials under real environmental conditions to establish protocols for the practical implementation of GO coatings in cultural heritage conservation.

## Figures and Tables

**Table 1:** Mean values of density and porosimetric parameters determined by MIP on natural and aged Euganean trachyte and Vicenza stone.

Sample	Bulk Density (g/cm^3^)	Apparent Density (g/cm^3^)	Open Porosity (vol. %)	Mean Pore Radius (µm)	Median Pore Radius (µm)	Total Pore Volume (mm^3^/g)
ET Natural	2.3506	2.6185	10.23	0.0792	0.0888	43.52
ET Aged	2.4154	2.7608	12.51	0.0942	0.1183	51.79
VS Natural	1.8004	2.4167	25.50	0.2216	2.4753	141.63
VS Aged	1.9459	2.8902	32.67	0.3421	2.0806	167.90

**Table 2:** Calculated D and G band parameters from the Raman spectrum of reference GO and GO–UV, as well as the pore and surface domains of the coated Vicenza stone samples.

Sample	Coating	Surface Feature	ID/IG Ratio	FWHM D Band (cm^−1^)	FWHM G Band (cm^−1^)
GO	reference		1.47	104	64
GO–UV	reference		1.48	113	63
VS Natural	GO	pore	1.79	105	63
		surface	1.44	77	57
	GO–UV	pore	1.78	92	62
		surface	1.80	101	67
VS Aged	GO	pore	1.56	90	57
		surface	1.72	86	56
	GO–UV	pore	1.56	94	57
		surface	1.22	66	51

**Table 3:** Mean values of water-related petrophysical properties determined on natural and aged samples of Euganean trachyte and Vicenza stone and the corresponding variation against the untreated surface for each type of coating.

Sample	Coating	Water Vapor Resistance Factor (Wet-Cup [65])	Variation Against Uncoated Sample	Water Absorption Coefficient by Capillarity (kg/m^2^ h^1/2^ [66])	Variation Against Uncoated Sample
ET Natural	uncoated	108.81 ± 2.15		0.328 ± 0.014	
	GO	124.40 ± 4.47	+14.3%	0.253 ± 0.018	−23.0%
	GO–UV	114.18 ± 7.32	+4.9%	0.167 ± 0.010	−49.0%
ET Aged	uncoated	110.56 ± 6.49		0.319 ± 0.016	
	GO	118.31 ± 11.20	+7.0%	0.241 ± 0.011	−24.5%
	GO–UV	119.68 ± 5.18	+8.2%	0.193 ± 0.014	−39.5%
VS Natural	uncoated	20.73 ± 0.54		8.027 ± 0.658	
	GO	24.15 ± 0.40	+16.5%	7.186 ± 0.160	−10.5%
	GO–UV	21.49 ± 0.58	+3.7%	7.073 ± 0.164	−11.9%
VS Aged	uncoated	20.89 ± 0.55		15.433 ± 0.508	
	GO	24.66 ± 0.73	+18.0%	13.118 ± 1.276	−15.0%
	GO–UV	21.39 ± 1.10	+2.4%	11.998 ± 1.295	−22.3%

## Data Availability

The original contributions presented in this study are included in the article/Supplementary Material. Further inquiries can be directed to the corresponding authors.

## Supplementary Materials

The following supporting information can be downloaded at: https://www.mdpi.com/article/10.3390/ma18061243/s1, Table S1: Calculated ΔE* and the average variation of L*, a*, and b* colorimetric components; Figure S1: HEX colour chart of samples following each surface treatment; Figure S2: Quantity of water absorbed by capillarity per surface unit area.
