# Supplementary material for: Performance Assessment of Graphene Oxide as a Protective Coating for Historical Stone
Source: Materials (Basel). 2025 Mar 11;18(6):1243. doi: 10.3390/ma18061243 (PMC11943864; doi:10.3390/ma18061243)
Supplement: Supplementary file 1 [file materials-18-01243-s001.zip › materials-3458454-supplementary.pdf]

Supplementary information:

Performance Assessment of Graphene Oxide as a Protective Coating for Historical Stone

Codrut Costinas [1,2], Liviu Cosmin Cotet [2,3,*], Lucian Baia [1,2], Naida El Habra [4], Luca Nodari [4] and Patrizia Tomasin [4,*]

[1] Faculty of Physics, Babeș-Bolyai University, M. Kogălniceanu 1, RO-400084 Cluj-Napoca, Romania; codrut.costinas@ubbcluj.ro (C.C.); lucian.baia@ubbcluj.ro (L.B.)
[2] Laboratory for Advanced Materials and Applied Technologies, Institute for Research, Development and Innovation in Applied Natural Sciences, Babeș-Bolyai University, Fântânele 30, RO-400294 Cluj-Napoca, Romania
[3] Faculty of Chemistry and Chemical Engineering, Babeș-Bolyai University, Arany Janos 11, RO-400028 Cluj-Napoca, Romania
[4] CNR-ICMATE (Istituto di Chimica della Materia Condensata e di Tecnologie per l'Energia), Corso Stati Uniti 4, 35127 Padova, Italy; naida.elhabra@cnr.it (N.E.H.); luca.nodari@cnr.it (L.N.)
* Correspondence: cosmin.cotet@ubbcluj.ro (L.C.C.); patrizia.tomasin@cnr.it (P.T.)

One of the most essential factors for a protective coating utilised in cultural heritage applications is the aesthetic variation caused in the substrate following its application. Table S1 displays the calculated average variations obtained for the colorimetric components in the CIEL*a*b* colour space, where L* is the lightness and a* and b* are the chromaticity coordinates in the red-green and yellow-blue directions, respectively, for a fresh GO coating and after a 7-hour UV exposure at λ = 254 nm. To offer a better image on the colour change induced by the GO coatings, a HEX colour chart is shown in Figure S1, which represents the colours of the same five points on the stone surface following the artificial ageing process, the application of the GO coating and its subsequent exposure to UV radiation for 7 hours.

Table S1: Calculated ΔE and the average variation of L*, a*, and b* colorimetric components for natural and artificially aged Euganean trachyte (ET) and Vicenza stone (VS) after GO coating and subsequent weathering through a 7h UV exposure.*

Sample	GO coating				GO-UV coating			
	ΔL*	Δa*	Δb*	ΔE*	ΔL*	Δa*	Δb*	ΔE*
ET Natural	-5.36 ± 1.14	2.26 ± 0.50	1.79 ± 1.60	6.30 ± 1.17	-9.11 ± 2.05	1.95 ± 0.51	0.01 ± 1.33	9.43 ± 2.00
ET Aged	-1.07 ± 0.77	0.30 ± 0.19	1.09 ± 0.37	1.66 ± 0.61	-4.82 ± 1.91	-0.01 ± 0.16	-0.70 ± 0.63	4.90 ± 1.94
VS Natural	-10.84 ± 1.32	2.25 ± 0.70	1.00 ± 0.88	11.16 ± 1.38	-14.53 ± 1.60	1.70 ± 0.73	-0.45 ± 0.79	14.67 ± 1.61
VS Aged	-4.29 ± 1.93	0.49 ± 0.44	1.71 ± 0.64	4.68 ± 1.97	-7.98 ± 2.22	-0.06 ± 0.40	0.26 ± 0.49	8.00 ± 2.22

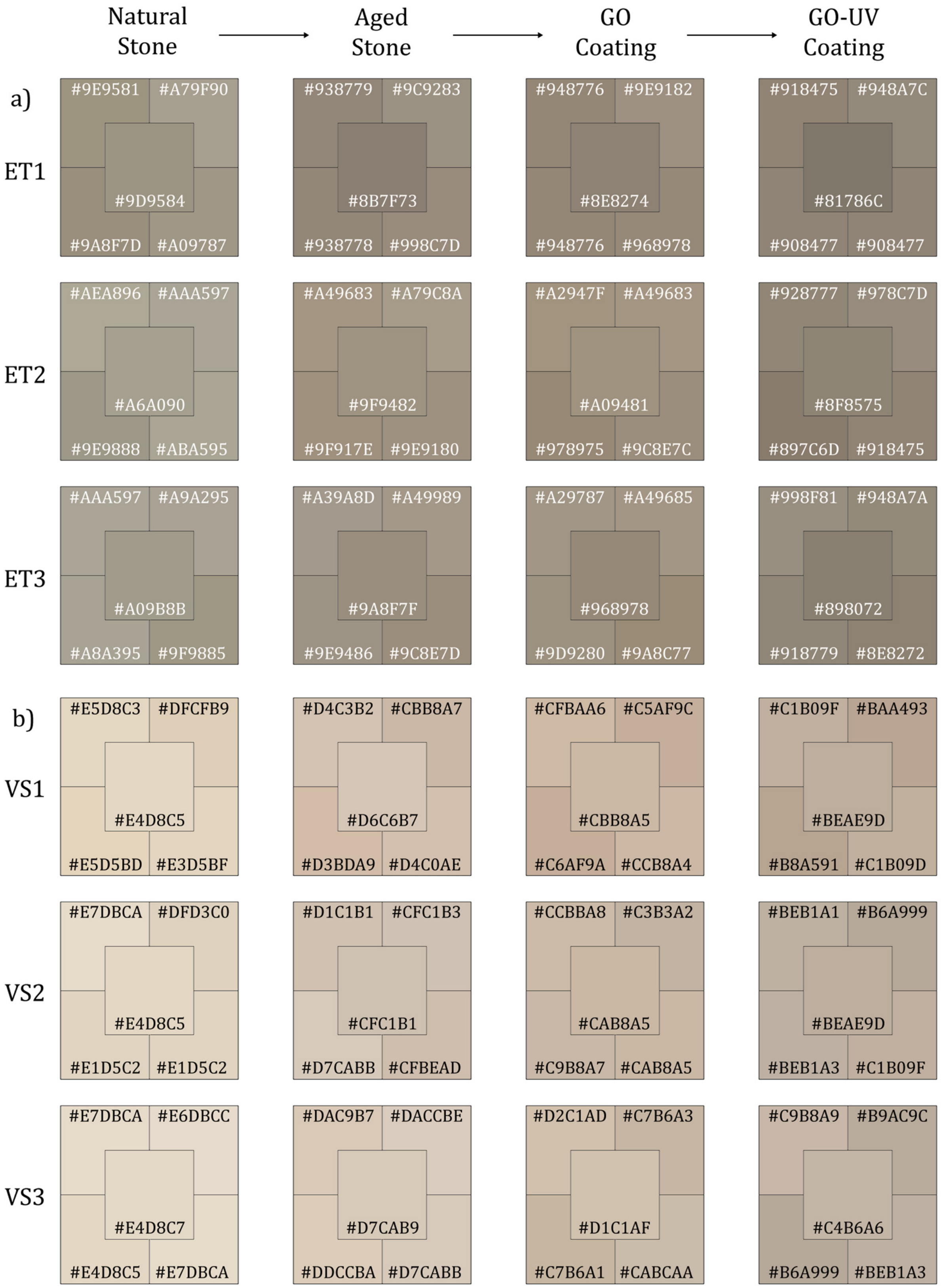

Figure S1: HEX colour chart of Euganean trachyte (a) and Vicenza stone (b) samples following each surface treatment. The same three samples of each stone type are represented in each column.

Capillary absorption curves are shown in Figure S2 for both the natural and aged stone samples of Vicenza stone and Euganean trachyte. For Vicenza stone the most notable variation is due to the artificial ageing process, which leads to a faster capillary absorption of water. Differences between the fresh GO and UV-exposed GO coating can only be noticed after 30 $s^{1/2}$ for the aged Vicenza stone samples. In any case, the same plateau is reached, regardless of coating. In the case of Euganean trachyte, however, the artificial ageing process does not cause any significant change in the capillary uptake dynamics, but the influence of the GO coating is very noticeable. The presence of the GO coating reduces the quantity of water absorbed, and the subsequent UV exposure further enhances the effect.

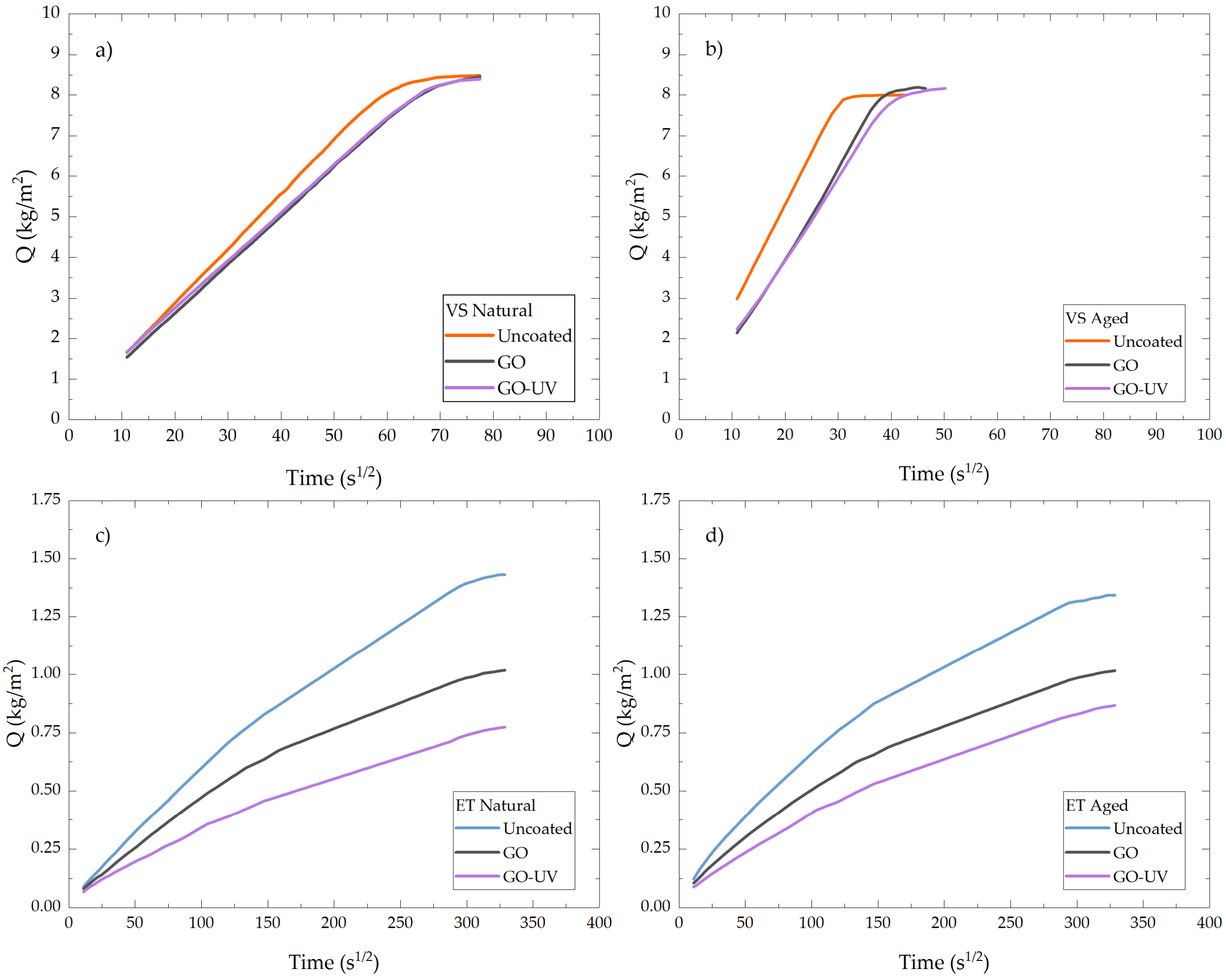

Figure S2: Quantity of water absorbed by capillarity per surface unit area, comparison before and after GO coating application, and after UV treatment, in natural and aged Vicenza Stone (upper) and Euganean trachyte (lower).
